# Habit Culture*Extract from a paper on Orthodontic Habit Culture, published in *International Journal of Orthodontia*.

**Published:** 1917-11

**Authors:** George Van Ness Dearborn


### HABIT CULTURE *

BY GEORGE VAN NESS DEARBORN, M.D., PH.D.

The science of habit, explanation of why habits form, may be stated very briefly for our present purpose as follows: The infant from birth (save when asleep) is so built (his nerves and muscles) that universal bodily movement is maintained. Movement-hunger, the "impulse to activity" keeps up this universal movement because the sensations arising thus are pleasant in themselves. Now, more or less by chance some of these movements prove especially gratifying and satisfying and therefore tend to be repeated over and over, and so to form habits. The sources of the satisfaction which underlies the formation of habits are nearly as various as the habits themselves. To name them would be to describe, almost, the motives of a young child's behavior, for almost universal is habituation.

Very few, even of biologists and psychologists, realize as yet how almost literally universal habit is in our move-

(* Extract from a paper on Orthodontic Habit Culture, published in *International Journal of Orthodontia.*)

ments (technically, altogether, called our behavior). But this is a fact and the fact has much importance. Only in an extremely few cases are our acts not in part already made easier by this process of habituation, namely, in truly voluntary acts.

Nature's object well attained, of course, is *economy;* economy of time, of energy, and of variation. Motions are done quicker, more easily, and more uniformly when they have been repeated until some degree of habituation has been secured. Practically our whole life is a habit-forming free. will; from birth and before the process is going on. During the first months after birth the child's education gets under way in the form of physiologic habits of eating, sleeping, etc., out of which as a base the more personal habits may gradually develop. This is nature's thrift, her practice of economy.

But a principle so basal and universal in action would be certain to work some harm as well as good. So, in the absence of supervision, some movements and acts are made habitual that are contrary to the inherent and natural rights of the child. He becomes, *pro tanto,* the victim of the vast rolling wheel of life, made slave by the "great chain wherewith," as Walter Pater says, "we are bound."

Among the many natural inborn rights of children scarcely recognized as yet in any effective way, are the rights to be first, as *healthy* and second, as *beautiful* as nature and nurture allow. These rights are as "inalienable" (as our national constitution says it) as the right to be *happy.* Indeed, the three are inseparable part and parcel of one portion of life, namely youth and childhood, "holiday," when already we have crossed one of life's borders and are still free from care. Health, beauty and happiness, these three, are inherent parts of normal childhood. A girl-body's beauty, as Maurice Hewlett says about "Sanchia's," "is but a poem written by God about her soul." But underlying this is the one only means by which any girl and any boy may arrive at length at personality, namely, the body itself, the material agent logically necessary in a material world. What parent, what nurse, what guardian would not give the children universally these—health, beauty and happiness? One of the surest and shortest methods is through psychological guidance.

The human face is the index of our personality, of our humanity itself. It is the most beautiful of all created things known to man. To preserve its normal beauty is a duty, a birthright due to every child. The outside of the human face is the at once most beautiful and interesting of all material objects whatsoever of which we know. The inside (that complex of organs and structure behind the "mirror of the soul") has more concern with health than has any other one portion of the body, and given health and beauty, happiness is not far away. This matter is really more important than the necessary brevity of its statement might suggest to you.

It is to habits of the inside and outside of the face, then, that your psychological interest in your children is to be especially directed: first, to prevent the formation of bad habits, and second, to bend them (when they are formed) down and out. No longer do we "break" habits or try to do so, lest in the fracture the delicate nerve system, all around them, be broken too. But we bend habits freely and finally displace them out of the child's mind and so out of his behavior.

Let us note, now, some of the classes of bad facial habits which it is the duty of the Post-Graduate School of this infirmary to discuss. I may be excused, probably, if I quote from a recent lecture on the psychology of habit:

"More or less arbitrarily we may consider four groups of kinesthetic habits especially, which lead to deformities of the mouth and of the dentition. In the first class we have the group which we may characterize as sucking movements, which involve the sucking of the thumb and fingers, fist, either lip, the tongue, or the clothing. Some of the worst cases of parodontic deformity come from the rather common habit of biting the clothing; boys especially get in the habit when young of biting the clothes and tugging on them. Perhaps more practically important than this, because commoner, is the use of the so-called "pacifiers," which really should be called something else, even defacifiers. A second group of actions is the biting-habit; biting either lip, the tongue, or the hands. Biting is a well-defined instinctive movement in most young mammals. Third, a group which requires less effort than the other two, namely, mouth-breathing, one of the commonest, I take it, of the causes of malocclusion. It appears to be always forced by a respiratory obstruction. The passage of air through the nose is normally, at least, as easy as it is through the mouth, so that no one with entirely unobstructed posterior nares breathes through the mouth. The fourth class of parodontic habits includes a very large number of arbitrary actions, including those of the busy hands in relation to the mouth, all sorts of tractions, distentions, etc., habits of many kinds that young children and sometimes older children get into, of handling their mouths in many various ways. I do not need to cite the numerous other causes of malocclusion, but there are at least thirty-five conditions which have been mentioned by witnesses as causes of this kind of deformity, diseases, maldevelopments, pure habits, too complex to even mention here, today. As important as any one thing is the use of "pacifiers," of many kinds, something that may be sucked, the sucking being, like virtue, its own and only reward. "Pacifiers" indeed! Men use them, you say? Men may use them (cigars), but not babies; it is a man's right, but in his case the defacement is relatively small.

To prevent the very existence of these unhealthful and disfiguring habits and such as these, is a psychological privilege, it seems to me, of every person who has any care of any child, from birth to puberty. It is also an obligation, and one not to be escaped without the bruising of one's con science.

Every child has a right to be as beautiful as nature allows, indeed it is a duty to be so. But "cosmetic values, of course, are very greatly disturbed by some of the muscular habits that children get into. If we compare the women with the children who sit before us in the trolley-cars, for example, in this respect, we can see the effects ahead as well as the causes behind. You can readily point out certain children who, when they become adults, will have badly deformed, and perhaps unworthy, faces, simply because they have been allowed to form the habit of holding their mouths, and so on, in various postures which are going to become fixed. How many good men are needlessly scared away from matrimony by this contrast between the middle-aged women whom they sometimes see and these women's children. Physical education should care for these conditions," but, out of inertia, it has not yet taken up systematically this task, much as it has done elsewhere for the betterment of the world.

The standard from which to judge whether a bad habit be developing or not is one of pure common sense. It needs only this, and no technical professional wisdom.

The practical method of preventing the formation of these habits of, on, and within the face, with the complicity often of the hands, is as simple yet as scientific as well it might be: *A self-interested understanding of the conditions involved, both immediate and future, an appreciation of the process, in short, and of its effects. Impression of the subconscious mind and hopeful encouragement, with the necessity of continued initiative of restraint.* In the way of pre vention, nothing can be done from without but this, nothing at least that is at all justifiable as a preventive method. On the other hand, perfect success by this method oftentimes is assured, at least in proportion to the intelligence and the promise of the individual child. One can not do more than this. Indeed, here in a way is almost a measure of the mortal's effective self-respect and of his proper educability. One can not do more. But in the long run it is safe to say that no child is going to allow her face to be disfigured, or her nutrition to be jeopardized, if she really understands fully her power to prevent such catastrophies. Why not let her and her brother understand it as fully as you can? And if no such habits impend, then the like philosophy of other bad habits which are fairly sure to be forming? As in psychotherapeutics, "mind-cure," there must be full comprehension and stimulating encouragement. These means will do much, very much more than you probably believe until you actually try them, to prevent bad habits of many kinds. You must secure in the child a continuous, if subconscious, attention to the task. The self-protective mechanism of the mind tends to accomplish the rest.

The sad familiar lines of Wordsworth sing in one's ears:

> "Full soon thy soul shall have her earthly freight,
> And custom lie upon thee with a weight
> Heavy as frost and deep almost as life."

But once formed, you ask, how may these disfiguring and often dangerous deformities of the face—so far functional and habitual, and not "grown in"—be cured? The means and methods have been hinted at already, but it may be made more explicit in seven points:

1. Not, at any rate, by literally "breaking" the habit. In adults, unless they be unstable, habits may be and often mercifully are, broken off short, although with raw and bleeding ends and edges of agony—the morphine habit notably. Habits can not be broken in this way in children, at least not after the age of two or three months. The problem here is a very difficult one, as difficult as childhood is from age.

2. The method then of bending bad habits down and out of a child's behavior is that again of psychological therapeutics, "mind cure." *A self-interested comprehension of the hard conditions involved, immediate and remote; with a vigorous impression and encouragement of the subconscious mind with the absolute necessity of continuous restraint.*

It is in this idea of the *incessancy* of the requisite personal control of the impulse to habituated action that the difficulty chiefly lies. James has emphasized in his discussion of habit what we all feel to be the very heart of the matter: "Each lapse is like the letting fall of a ball of string which one is carefully winding up; a single slip undoes more than a great many turns will wind up again." Hence the need of employing sentinels and guards that never sleep and which are to be absolutely relied upon; for the impulse to activity knows no such things as inattention, but every moment watches for an opportunity in proportion to its habituation.

No other guard whatever serves or can serve this supreme watchfulness save the (subconscious) mind itself, loyal protector always, without a lapse, of the best interests of the individual so far as they are impressed upon it by the freer intelligence and through the stringent orders of the emotional, dynamic phase of the child.

It is like inducing an individual to be good or to be kind or to be gentle, or an active youngster to be quiet. It requires continual effort from within. This, however, is an education in itself. This, indeed, is pretty much of the whole matter: a true and full understanding of the habit-trap and its injuries, and of the ease and certainty of escape provided the child consciously and subconsciously really and sincerely wishes to be relieved of it. It all depends on continuous voluntary initiative founded in enthusiastic confidence and determination.

3. Encouragement and confidence are essential, for the average child has as yet little self-reliance. It is part of his very childhood not to have it, and the wholly self-reliant child is already a man or a woman.

4. Another essential feature of the educative habit-bending methods is that one habit only at a time, for the most part, may be attacked. The reason for this is the wise old adage of our grandmothers:

> "One thing at a time and that done well
> Is as good a rule as any can tell."

In the physiologic terms of psychology, this is the principle of the "final common path," or, in this case, of cortical resultants.

5. The depression and the discouragement of fear and worry are wholly out of place in this inherent bending of a child's subtle and sensitive mind. Fear and worry block the will and hinder it, and lower its vigor as they do its spontaneity. Fear is wholly incompatible with the best results in such influence as this, and that wholly aside from the danger of imparting a lasting shock to the brain of a child.

This necessity of avoiding fear and worry in the child, eliminates serious threats of future punishment. It rules out, too, all harshness and severity. This is not that kind of a problem at all. You do not threaten a boy or abuse him for a too long convalescence from tubercular hip disease; neither should you in this case.

Punishment is in fact outside this whole matter unless, be it first, of a mild sort; second, highly successful; and, third, free from all terror. Corporal punishment would be an absurd factor in a process of subtle reform from within outward such as we are discussing. You must impress the mind rather than the soft part of the back, and so in principle all through. The whole Freudian doctrine of repression and rankling and nervous exhaustion comes into this problem when early shock or fear has been incurred. The far-reaching evils thus made possible would be worse indeed than the original bad habit; in this case, undoubtedly, the remedy is infinitely worse than the disease.

6. Busy normality of the nerves and muscles and glands is an important adjunct, and often a necessary preliminary, to habit-bending when the habits are of serious import to the child.

Back to the farm, even in winter. Months of freedom and plain outdoor living among the beautiful hills and rapid streams and the maple groves of Vermont or along the satiating Acadian seashore—herein lies invigoration. In practice sometimes only in this manner is the mechanism of nerves, muscles and glands given its normal vigor to recognize habit abnormality; to refuse its allurements; and by its inherent powers of self-protection to bend it out of existence again.

In many cases of deranged moto-sensory habit an active and conscious normalizing thus procured, in intelligent children, is enough almost in itself for a cure.

7. In other instances it must be admitted, the skilled orthodontist or even the surgeon finds his wits well taxed to make things right. But the way of ways is prevention rather than cure, a system for guiding the daily inclination of the "twig" rather than the difficult and tedious and even dangerous back-bending of the "tree." We call prevention of this kind by a familiar special name, prophylaxis, but more properly, here, it is psycho-prophylaxis. Prophylaxis is scarcely born as yet in either medicine or dentistry. When, however (before many decades), it has become part of the common mind of all the people, how crude, how extravagantly wasteful, will seem the neglect of the present day! Nothing less than shameful to our boasted human intelligence is the collossal wastefulness of happiness and life and money in this respect. What is it then which many of us are so much in a hurry to obtain that we can not stop to live? We should prevent disease, altogether, and deformity.

It is fortunate, indeed, for us all that this bending of a habit itself becomes habitual and so ever easier and easier.

"To him that hath shall be given." "God helps those who help themselves." Thus the habit of bending any particular habit soon becomes habitual, as do the habits of close study, of taking sufficient exercise, of cold morning baths, of giving your loving wife her inevitable last word, or of any other habit hard, often "beastly" hard, at first to compass. So the child by trying at last succeeds, and more easily, often, than at first seemed possible. And by trying, whatever his years, he becomes a personality—a man—captain of his soul.

Nothing is finer, let us be sure, or few things, at least, in this wondrous life of ours than this slow, hourly mastering of ourselves, a bit by bit, day after day. Then at length we begin to appreciate the privilege of being conceived and born into even a brutal world, an experience often harsh and worrisome and sad, often cruel, even, to a wholly unbelievable extent, but always, notwithstanding, full of an almost inexplicable joy arising to a large extent in our own dominance of ourselves.

This joyousness of satisfaction has its most familiar symbol in the crushing of inexpedient habits—links in that great living chain with which life forever binds itself, but through which alone human personality is conceivable.

